# Impact of the COVID-19 pandemic on health care and daily life of patients with rare diseases from the perspective of patient organizations – a qualitative interview study

**DOI:** 10.1186/s13023-023-02771-w

**Published:** 2023-06-21

**Authors:** David Zybarth, Maja Brandt, Christine Mundlos, Laura Inhestern

**Affiliations:** 1 Department of Medical Psychology, University Medical Center Hamburg-Eppendorf, Hamburg, Germany; 2 Allianz Chronischer Seltener Erkrankungen (ACHSE) e.V, Berlin, Germany

**Keywords:** Rare disease, Healthcare, COVID-19, Sars-CoV-2, Patient organization

## Abstract

**Background:**

During the COVID-19 pandemic people affected by rare diseases (RD) or caregiver of affected children have faced additional challenges. The pandemic has affected physical and mental health, social life and has led to financial consequences. Our objectives were to identify the impact of COVID-19 (1) on health care and (2) on daily life and participation of patients with RDs or caregivers from the perspective of representatives of patient organizations. Moreover, we explored their perspective on experiences of pandemic stress and resources during the pandemic.

**Results:**

We conducted 18 semi-structured interviews with representatives of patient organizations (e.g. chairperson, members of the steering committee), who were asked about the experiences of their members. The interviews were transcribed verbatim and analyzed using the framework approach. We contextualized our findings on the basis of the International Classification of Functioning, Disability and Health (ICF) model and adapted it according to identified subthemes. Patients and caregivers were confronted with aspects of pandemic stress such as lack of information, access and information regarding vaccination and being a risk group for COVID-19 infection. Physical and mental functioning was reported to be negatively impacted. Lock downs and contact restrictions led, e.g., to increasing lack of nursing services or lack of necessary informal support. Participation e.g. in social life and work was reduced. Health care services including medical care and supportive care as well as additional therapies were disrupted and greater effort was necessary to organize care. According to participants, central resources were informal support networks, digitalization, patient organizations and individual characteristics.

**Conclusions:**

Our study highlights the consequences of the COVID-19 pandemic on the situation of people affected by RDs and caregivers. Contextualization of the results into the biopsychosocial model reinforces the impact of the pandemic on health care as well as daily life and participation. Major challenges and difficulties were experienced during lockdowns and contact restrictions. Depending on the risk of an infection with COVID-19, certain patient groups were still isolated and reduced social contacts or still followed strict hygienic measures (e.g., wearing medical masks). Future pandemic control measures, e.g. on lockdowns and closing facilities, should consider the challenges of people with RDs and caregivers of affected children.

**Supplementary Information:**

The online version contains supplementary material available at 10.1186/s13023-023-02771-w.

## Introduction

Approximately 300 million people worldwide are affected by at least one of the over 6000 different rare diseases (defined as 5 people or fewer of 10.000 being affected) [1]. People affected by a rare disease (RD) experience challenges in healthcare due to long periods until assured diagnosis, centralized expertise of medical care or lack of effective treatment [2, 3]. The disease and its symptoms can impact daily life and participation in society [2, 4]. Depending on the disease, multiple organ systems can be affected and physical, cognitive or functional limitations can occur [5]. Studies on mental burden indicate higher levels of depressive symptoms, anxiety and reduced quality of life [6, 7]. At the same time, people with RDs do not feel well supported, especially apart from the medical aspects of their disease [8].

A resolution by the United Nations Assembly describes and highlights the specific challenges of people affected by a RD e.g., participation in society or access to quality health service and stresses out the consequences of the COVID-19 pandemic as it might impact access to health care and the situation of people living with RD[9]. Particularly in times of strict lockdowns, patients depending on functioning healthcare system have been affected [10]. The pandemic has affected their physical and mental health, social life and has led to financial consequences [10]. People affected by a RD showed higher levels of depression and anxiety compared to the general population [11]. Depending on the diagnoses, COVID-19 was associated with higher risk leading to increased necessity for social isolation and contact reduction [12]. Recent studies also identified consequences in medical care and research: health care and necessary examinations or treatment could not be ensured continuously [12, 13]. Access to essential and elementary health services was reduced [14]. In Italy, during the first phase of the COVID-19 pandemic, prevalence of newly diagnosed people was lower compared to 2018 or 2019 indicating difficulties in maintaining necessary diagnostic measures [15]. Clinical research on cystic fibrosis was disrupted due to restrictions of clinics and difficulties in maintaining personnel and medical supply [16].

As professional support offers are lacking or are not regional available, peer support is an important aspect and resource of support. Even before the COVID-19 pandemic, patient organizations played an important role in supporting people affected by a RD [8, 17]. They provide guidance, information, emotional and social legal support and peer support. Recent developments have strengthened involvement of patient organizations in research as partners reflecting patient experiences [18–20]. They represent the patients’ perspective and needs of their members in research approaches, e.g., medical or treatment innovations. During the COVID-19 pandemic, patient organizations maintained their availability for their members and patients respectively caregivers [10, 21] and had a great insight into the situation of people affected by a RD.

Our main objective was to identify the impact of COVID-19 (1) on health care and (2) on daily life and participation of people living with RD. Therefore, we conducted a qualitative interview study to assess the perspective of representatives of patient organizations on the impact of the COVID-19 pandemic on their members and the RD population they represent. Moreover, we explored their perspective on experiences of pandemic stress and resources of their members during the pandemic.

## Methods

This interview study is part of a study on the impact of the COVID-19 pandemic on people affected by RDs respectively caregivers of affected children using perspectives of patients and caregivers (qualitative and quantitative study design) and representatives of RD patient organizations. The presented interview study focuses solely on the perspective of representatives. The reporting follows the current practice guideline COREQ (consolidated criteria for reporting qualitative research).

### Participants and recruitment

We aimed at including representatives, mainly active members ((e.g. chairperson, steering committee, group leader), of patient organizations as we assumed that they would have a broader view on the experiences of those people they are representing (certain RD groups; patients, caregivers). We identified potentially eligible participants via our cooperating partner, the “Alliance for rare chronic diseases e.V.” (ACHSE), an umbrella organization for RD patient organizations in Germany. The ACHSE disseminated basic study information via their E-mail distribution list of member organizations and sent two reminders. Additionally, randomly selected member organizations were additionally invited for participation by study team members. We started consecutive sampling strategy and included interested representatives of patient organizations into our study [22]. To reduce a gender bias and a bias regarding patients compared to caregivers, we switched to a purposive sampling strategy by mailing those patient organizations with male persons as chairpersons or in the steering committee and patient organizations focusing on diseases which mainly affect children. In case of interest in study participation, detailed study information and consent form were sent to participants via E-mail. In a few cases, reasons for non-participation were given by persons who were asked to participate in the study, which was most often that they had no time or no personnel resources. In most cases, there was no feedback in the case of non-participation.

Our aim was to include 15–20 participants as it was indicated a sufficient number to reach theoretical saturation [23]. After 18 interviews were conducted, we did not include any further participants as the point of saturation based on the preliminary assessment of interviews was reached.

### Data assessment

We conducted semi-structured interviews using an interview guideline including mostly open questions with the possibility to ask deepening questions (Table 1). Moreover, basic data on the interviewee and the patient organization he or she represented were collected. The questions were developed based on previous literature of the impact of the COVID-19 pandemic on the RD community and suggestions from the ACHSE. We conducted a pilot interview with a representative from the project cooperation partner, the ACHSE, which was excluded from the analyses, as the ACHSE was involved in the development of the guideline. All interviews were conducted in German by telephone by DZ, MB or LI (all psychologists and experienced in conducting qualitative research). The interviewees were informed about the study, the study aims and about the basic occupational details of the interviewers (researcher, psychologists).

**Table 1:** Overview of interview questions relevant for the objectives

Topic	Question
Introduction	Do you have any questions before we start the interview?
Sociodemographic data and basic characteristics of the patient organization	
Negative impact of COVID-19 on the people represented by the patient organization	Which consequences and changes due to the COVID-19 pandemic did you notice in your RD community since the beginning of the pandemic? (in health care, in daily life) What were/are the key stressors you observed or noticed?
Resources and Needs	What positive aspects did you notice? What aspects have helped or would have helped people with RD to reduce or manage stressors? What kind of support was lacking? How would the best possible care and support would have looked like?
Closing	Is there anything else you were not able to report in the context of the previous questions which you consider important to understand the situation of the people represented by the patient organization?

Interviewees provided written informed consent. All interviews were audio-recorded and transcribed verbatim. Field notes were taken by the interviewers. Transcripts were not handed back to interviewees for correction.

### Analysis

We analyzed the interviews using the framework approach for analyzing qualitative data [24]. First, LI read all transcripts and initially assigned preliminary codes. First themes were retrieved from the data and codes. The themes were reviewed against the background of all identified codes and discussed within the study team (LI, MB, DZ). The ICF model was identified as a suitable framework for the data. The model allows for a multidimensional approach to assess health and particularly addresses participation and chronic diseases [25, 26]. Accordingly, themes and codes were contextualized and integrated into the domains of the biopsychosocial model of the ICF.

All analyses were conducted using MaxQDA software (version 20) for qualitative analyses [27].

### Patient and public involvement

Representatives of the ACHSE as umbrella organization of patient organizations for RDs were included into the planning of the study from the early beginning. Moreover, they supported participant recruitment. Results were presented to patient representatives and discussed. CM was additionally included as author according to the criteria for authorship based on the International Committee of Medical Journal Editors (ICMJE) [28].

### Characteristics of participants and represented patient organizations

18 interviewees representing 15 different patient organizations for RD participated in our study. The majority was female (n = 13), mean age was 54 years (SD = 11 years). Eleven participants had more than 10 years of a school education. The majority of interviewees was first, second or vice chairman (n = 13) of the patient organization. Almost all participants (n = 14) were involved in the patient organization for more than eight years and had held various functions since their active involvement. The number of members or estimations of members of the represented patient organizations ranged from 150 to 7.500 members (including patients, relatives, health care professionals).

## Results

**Fig. 1:**
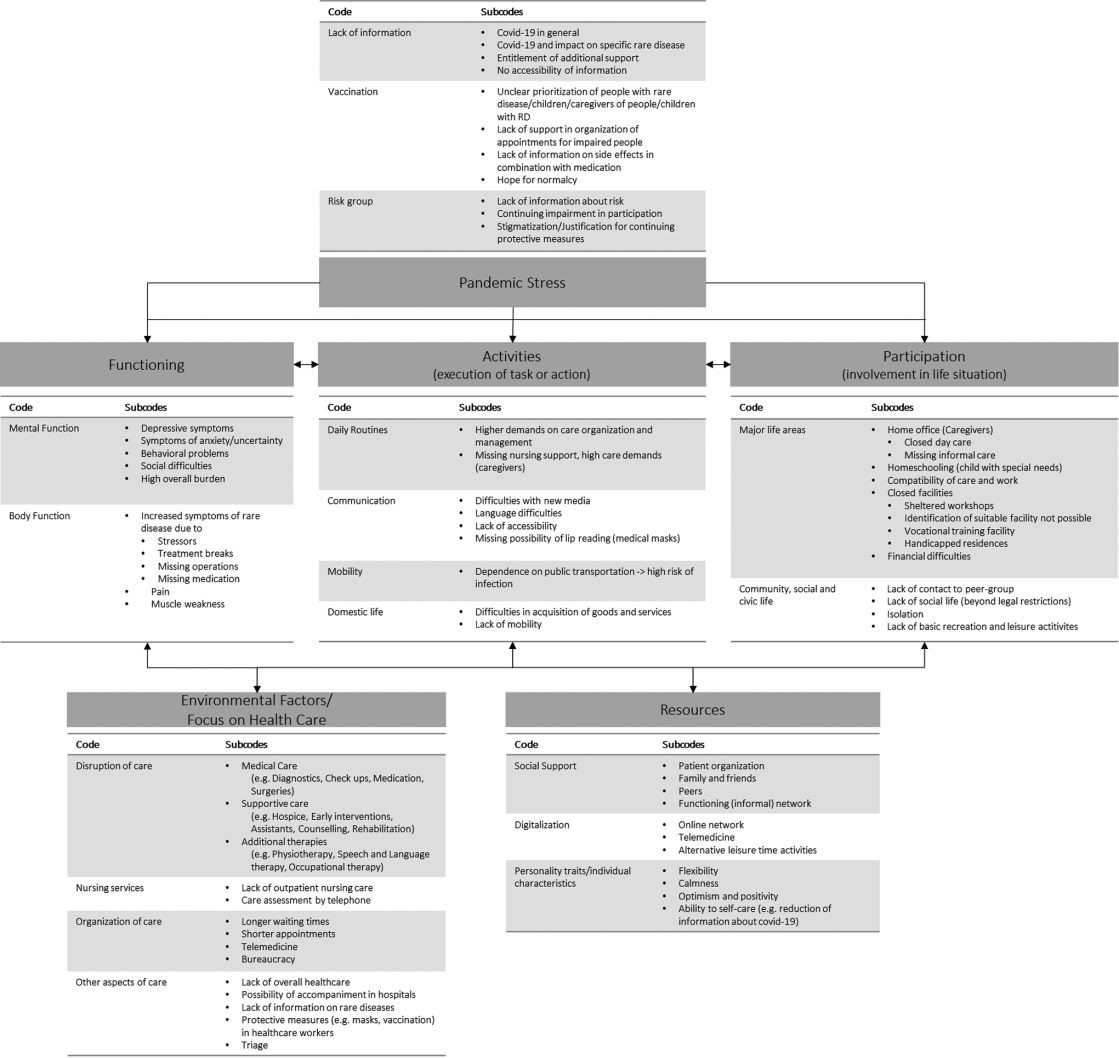
Themes, codes and subcodes contextualized on the basis of the bio-psycho-social model of the ICF [27]

Five key themes were identified: pandemic stress, functioning, activities/participation, healthcare and resources. We contextualized the themes into the biopsychosocial model of the ICF [27] (Fig. 1). A detailed table of themes, subthemes and example quotations is presented in the Supporting Material (Table S1).

### Pandemic stress

Categories that were identified regarding to pandemic stress were lack of information, vaccination (information and access) and risk group. It was reported that besides information on COVID-19 in general, information of COVID-19 in combination with the specific RD was lacking. Moreover, information about the eligibility for additional support (e.g., extra financial support for hygienic equipment) has not reached all of those in need. Information were not routinely accessible. As most specific information was provided online, several barriers were described (e.g., readability for impaired people, difficult language). Whereas some interviewees reported similar experiences regarding vaccination for their members compared to people without RD (e.g., difficulties to get an appointment), it was reported that in some cases the prioritization group was unclear (e.g., affected children, caregiver of children or diagnosis of a RD in general). According to the interviewed representatives of patient organizations, particularly older and impaired people with RD without an informal network supporting them had difficulties to get vaccinated. Other aspects mentioned were missing information regarding side effects of the vaccination in combination with medication and hope for normalcy after vaccination of the general public.

### Functioning

Representatives of patient organizations described mental and physical consequences for their members. They mentioned symptoms of depression and anxiety among their RD community as well as social difficulties and high overall burden. Behavioral problems were described in affected children. Caregivers of affected children were mentioned as particularly burdened, particularly during phases of lockdowns including closed schools and care facilities.

With regard to physical functioning some aspects such as an increase of symptoms of the RD or additional symptoms such as pain and muscle weakness were described.

### Activities and participation

According to representatives of patient organizations, people affected by a RD and caregivers experienced major changes in their daily routines. Care and disease management needed higher efforts. Reported reasons were closed facilities or restructuring health care e.g. due to hygienic measures. Moreover, participants reported that informal support network (e.g., due to contact restrictions or fear of infection) and nursing support were reduced (e.g., due to lack of nurses). Particularly, caregivers were reported to have experienced higher demands. Whereas lockdown regulations were terminated for the general population in Germany at the time of the study, it was described that some members still had difficulties regarding their daily routines due to a lack of supportive care and fear of infection.

Another activity which was reported to be impeded by the COVID-19 pandemic and associated regulations was communication. Difficulties with new media and digitalization, language difficulties and lack of accessibility hindered online communication. Obligations of medical masks reduced possibility of lip reading in contacts in person.

Participation in job and education was limited at least during the time of lockdowns, including closed facilities, home-office regulations and homeschooling. It was reported that in times, where conventional school and kindergartens had already opened again, some sheltered workshops, integrative kindergartens, vocational training facilities or handicapped residences were still not back to the pre-COVID-19 opening hours or were still organized in altering shifts (e.g., weekly). Simultaneous home-office, home-care and home-schooling of children was described as particularly challenging for parents of children with special needs.

Community and social life was characterized by a lack of peer-contact and lack of social life (beyond legal restrictions) leading to experiences of isolation. Moreover, basic recreational and leisure activities were abandoned during lockdowns and have not yet resumed by some members.

### Experiences in health care

As many environmental factors changed during the pandemic, presented results focus on changes in health care reported by the participants. According to representatives of patient organization, health care services including medical care and supportive care as well as additional therapies were disrupted. Moreover, home-nursing services were temporarily cancelled and disrupted (e.g., due to lack of personnel). In case of an application, the case assessment initiated by the nursing insurance was undertaken by telephone during contact restrictions, which sometimes led to incorrect assessments. Some participants reported that their members experienced more challenges in care organization. E.g., higher waiting times, shorter appointments and bureaucracy were mentioned. Other aspects regarding changes on health care for patients with RDs were described (e.g. lack of overall healthcare or limited possibilities for accompaniment in hospitals; see Fig. 1).

### Resources

The representatives of patient organization identified informal social support from family and friends, peers and the patient organization as a central resource for people with RDs. Digitalization was described as a possibility to stay in contact with the social network, enabled appointments with health care professionals and sometimes provided an opportunity for alternative leisure time activities. Furthermore, personality traits and individual characteristics such as flexibility, calmness and optimism were reported as resources.

### Suggestions

Study participant retrospectively suggested how the situation of affected people or parents could have been improved during the COVID-19 pandemic. Concerning medical care, ensuring continuity of and access to care was central. According to the participants, home visits would have been a way to maintain care if people were not sufficiently mobile (e.g., high risk of using public transportation, incapability to drive due to physical limitations). Organization of medical care could have been improved by using synergies, e.g., between specialists and general practitioners, or by implementing a central care coordination. To generate evidence-based information assessment of side effects caused by vaccinations or symptoms of COVID-19 in specific RDs, the use of registries would have been a possible way.

Concerning supportive care, continuity was a central aspect (e.g., assistance, nursing service, additional therapies). Furthermore, necessary additional support could have been proactively offered (e.g., by authorities or health care providers). Digitalization was perceived as a way to provide support. However, the exclusive use of digital offers would exclude specific patient groups and was not perceived appropriate for all concerns.

The general organization of the measures to combat the pandemic was criticized as bureaucratic. Reduction of bureaucracy, including more flexibility for individual cases as well as more transparency and clarification of current regulations and provision of infrastructure, medication and masks was suggested by participants. Moreover, participants thought that the visibility of at-risk groups such as people with RDs could have been increased in politics and society.

## Discussion

Our study highlights the consequences of the COVID-19 pandemic on the situation of people affected by a RD and caregivers. Contextualization of the results into the biopsychosocial model reinforces the impact of the pandemic on health care as well as daily life and participation. According to representatives of patient organizations, major challenges and difficulties were experienced by their members during lockdowns and contact restrictions. However, depending on the individual risk of an infection with COVID-19, certain patient groups were still isolated or reduced social contacts or still followed strict hygienic measures (e.g., wearing medical masks).

The COVID-19 pandemic has affected society as a whole. The population in Germany and many other countries experienced lockdowns, the closure of schools and childcare facilities. People living with RDs have already experienced specific burden and challenges before the pandemic. The results indicate that the burden and needs were high during the pandemic and that particular vulnerable subgroups among the RD community such as older people, people with impairments or parents of seriously ill children were especially affected.

Consistent with previous findings [29], our findings indicate that the COVID-19 pandemic and its consequences intensified the challenges of patients with RDs and caregivers. Lack of information and concerns about necessary medical care, might have led to high uncertainty and aggravated symptoms of anxiety or depression. After some months, structures in the clinics and medical facilities reorganized, still, there was a backlog of appointments as these were cancelled or postponed by clinics temporarily or by patients due to fear of infection.

As activities and participation were impaired, psychosocial burden additionally was high and, at least in parts of the RD community, has not gone back to pre-pandemic levels at time of the interviews. A specific group suffering from social restrictions were children in general [30]. Conventional schools and kindergarten were closed during lockdown periods, leading to phases of home-schooling and lack of peer contact. The school closures have impacted children’s mental health and education, implying that policymakers, teachers and researchers should inform concepts for future crisis [31]. Still, our results suggest that some care facilities for children with special needs had even longer periods of limited hours e.g., due to difficulties to implement hygiene concepts, and, hence, participation was limited even longer. As interviewees reported, some children showed more symptoms of behavioral problems, e.g., in case of preexisting autistic symptoms [32] or in case of social isolation. Caregivers of affected children experienced high overall burden. In parts, multiple stressors such as care organization, nursing tasks, home-schooling and home-office seemed to last even after legal restrictions had been removed. These aspects support the high need of school/kindergarten concepts children with special needs as well as of low-threshold psychosocial support offers to validate and unburden vulnerable groups [33], such as patients with RDs or caregivers of chronically ill children.

One possibility to provide psychosocial support that was quickly implemented in many contexts was telehealth option. Particularly among the RD community, this might be a promising approach to prevent long distance travels. However, routine psychosocial support for people with RD has not been implemented yet in Germany, neither in presence nor as telehealth solution. Support regarding psychosocial needs is often provided by patient organizations [34]. Besides psychosocial care, home healthcare support has also been discussed to unburden families in need [32].

### Strengths and Limitations

Patient organization have provided support during the COVID-19 pandemic. Through the perspective of their representatives, the description of experiences of those affected is condensed and based on requests by and exchange with members from their RD community. Firstly, as many of the representatives were affected themselves or caregivers, particularly those aspects, that concerned them themselves, might be overrepresented in the interviews. Secondly, experiences of people with RD not related to patient organizations might have been missed. Thirdly, from a large number of patient organizations, we only included participants from 15 different organizations. We stopped recruitment when no further themes occurred. However, we might have missed aspects.

Lastly, our data collection was in summer to autumn 2022. This not only allowed it to describe experiences during the first phase of the pandemic, but also to include impressions from the time after general social restrictions were removed. Still, recall effects of certain phases among the COVID-19 pandemic may lead to biases.

## Conclusions

RD community is a vulnerable patient group suffering during the COVID-19 pandemic due to pandemic stress, disruption in health care and daily life. During the pandemic, participation was impaired for adults and children with RD as well as caregivers. Understanding the situation of affected people can help to design the health care and support system to meet the needs of this vulnerable group and might sensitize the society. Future pandemic control measures, e.g. on lockdowns and closing facilities, might consider the challenges of people with RDs and caregivers of affected children.

## Electronic supplementary material

Below is the link to the electronic supplementary material.

Supplementary Material 1

## Data Availability

All data relevant for the results are presented within the manuscript. Interview transcripts are not publicly available due to the sensitive nature of the data.
