# Supplementary material for: Impact of the COVID-19 pandemic on health care and daily life of patients with rare diseases from the perspective of patient organizations – a qualitative interview study
Source: Orphanet J Rare Dis. 2023 Jun 21;18:154. doi: 10.1186/s13023-023-02771-w (PMC10283205; doi:10.1186/s13023-023-02771-w)
Supplement: Supplementary file 1 — Supplementary Material 1 [file 13023_2023_2771_MOESM1_ESM.pdf]

Table S1. Themes, codes and example quotations.

Theme	Code	Subcodes	Example quotation[1]
**Pandemic stress**	Lack of information	• General aspects<br>• Entitlement of additional help<br>• Specific rare disease and corona	• Because there was a great deal of uncertainty and there was a lack of possibilities to receive information. You could find information about everything: how much short-time work benefits do you get, how much money do [large companies] get, how are companies supported? But simple questions, how do we organize nursing services, what measures do we have to take and how do facilities support us with very banal things? Mask protective equipment, gloves etc.<br>• In the beginning it was very chaotic. Nobody knew what it was, what the consequences would be [in case of COVID-19 infection and rare disease) and how do I deal with the children.
	Vaccination	• General aspects<br>• Vaccination for children<br>• Insecurity<br>• Organization of appointment<br>• Priorization<br>• Burden due to vaccination opponents	• And the parents jittered, what about our children. There were no vaccinations for children yet. Everybody was happy, when the first vaccinations for children came and then the considerations – should I have my child vaccinated or not?<br>• That [organization of appointment for vaccination] was really complicated. And for someone who doesn't have a laptop and such, family members got it for grandma or grandpa. That was difficult, or trying it themselves on the telephone, that was damn difficult<br>• What about us, we have pre-existing conditions, actually we should have gone very high [in the priorization group for vaccinations], well, but then they put us in the fourth or third group
	Risk group	• General aspects<br>• Following rules	• It started with unbelievable fear at the beginning [of the pandemic] and in some cases really complete self-isolation, because it became clear relatively quickly that COVID also has an effect on the heart, so that people were very afraid of getting infected.<br>• We still have some members who are extremely careful. It's getting better, but there are still some who are extremely careful, who also wear masks everywhere [...].
**Functioning**	Mental burden	• Anxiety<br>• Depressive symptoms<br>• Helplessness<br>• Insecurities<br>• High overall burden	• You are just afraid, you do not want to have any contact and do not even notice that you are just sitting at home and no longer have any contact to the outside world.

Theme	Code	Subcodes	Example quotation[1]
			• Man hat einfach nur die Angst, man möchte keinen Kontakt haben und man merkt gar nicht, dass man dann zuhause sitzt und ja, und keinen Kontakt mehr nach außen hat.<br>• I know about one family – they almost lost their place in assisted accommodation, because their child fall into depression. She got bullied and her mother decided to have her home for medical reasons and then she was threatened with having her place taken away in the accommodation.<br>• When all appointments were cancelled, medical appointments. Our people were completely helpless.<br>• […] with the beginning of the pandemic there was a high level of uncertainty […]<br>• […] concerns and distress was mainly experienced by trying to not get infected. You are already punished with your disease. There is no cure. The only thing you can try is to stop a more severe course of disease. And if I can't stop it because the surgery gets cancelled, that is a huge burden.
	Worsening of symptoms		• Then there are the pain symptoms getting worse and the muscles getting weaker.
**Activities**	Daily Routines	• Higher demands on care organization and management<br>• Missing nursing support, high care demands (caregivers)	• At first [in the pandemic] it was difficult, many did not dare to visit their therapists or doctors, because they were afraid of getting infected. There is one clinic for the whole of Germany, which is the only clinic where detailed diagnostic of our disease is carried out. This department got closed meanwhile, because they needed the personnel to care for COVID-19 patients. This lead to extended waiting times - up to one year.<br>• Health care deteriorated enormously since at first of the pandemic nursing staff didn't come anymore, the patients did not receive necessary care. Of course, that was massive.
		• Break down of informal support network	• From one day to another everything was gone, which has brought trouble to the one or the other, because daily support network, Grandma, Grandpa stepping in – they were in the group worth protecting. Which means, this network falling apart. This means our families were in the dilemma of job, day care and in general this

Theme	Code	Subcodes	Example quotation[1]
			organization of life […]. This network that you build up was gone from now on. That was a huge problem.
	Domestic life	• Difficulties in acquisition of goods and service<br>• Lack of transportation	• Partly, families and friends brought that [goods] and put it in front of the door. Or some families used a pick-up and delivery service […]
	Communication	• Difficulties with new media<br>• Language difficulties<br>• Lack of accessibility<br>• Missing possibility of lip reading (medical masks)	• Not everybody is well trained and not everybody has the technical ability to use technique solutions for meetings. That lead to social isolation, even stronger than before.<br>• We had difficulties since our children or affected people are visually and hearing impaired. That is a huge problem, that too many information got lost when everybody was wearing masks. They couldn’t see the mouth; they couldn’t hear properly. That is a problem which was not addressed properly depending on the facility. In some facilities transparent masks were used, but often it was not, which was leading to major problems.
Participation	Community, social and civic life	• Lack of contact to peer-group<br>• Lack of social life (beyond legal restrictions)	• They [children with the disease] do not have a party every weekend or endless friends or just step by for a short visit at Grandmas and so on. So organized weekend- and holiday-activities and camps are extremely important for these young people. If these can’t happen, it means, that they can’t meet their peer group and don’t have that, where they actually feel comfortable. […] And I think that was a special hard situation.<br>• During summer months, when actually everyone could go out and meet, there still was this fear, because you saw the others didn’t really protect themselves. We have tried to meet outside in the summer, but they [members of the organization] cancelled us, one person might have come. You can tell, the people have withdrawn completely.
		• Isolation	• Another effect was, that there actually were people, which more or less never left their home, isolated themselves extremely in winter and, they, if at all, only met people outside in summer.<br>• During the course of the pandemic you really notice, the people became lonely, because they don’t dare to go outside, because they always said to themselves to be careful, because they are risk patients. They try to go as little as possible among people.

Theme	Code	Subcodes	Example quotation[1]
		• Lack of basic recreation and leisure acitivites	• I mean it is also the case that there are people, who listen to the radio, but if their care worker isn't there, they can't turn on the radio. That's as far it goes.<br>• I think, it was less [leisure activities] for everyone, but for those represented by us [the patient organization] it was even worse, because they are already fewer sports facilites, because there are fewer choirs or whatever activities they could do with these disabilities. It that sense, I do thank, that it hit them even harder.
	Major life areas	• (Home) Office<br>• Compability of care and work<br>• (Home)-Schooling<br>• Closed facilities<br>• Financial difficulties	• We were kind of pushed into homeoffice. We weren't asked whether we want it or not. I think it was similar for everyone, but you didn't dare to ask, but you were just generally afraid.<br>• And when he was allowed to do back to work, work was organized in shifts. And this shift work was maintained for over a whole year. That means, every other week there was the challenge of daycare, I had the situation to look after him and to keep him busy and so on. […]<br>• [….] When you have to work from home or something like that, you can't do that with our children [represented in the patient organization]. These children need us, every minute keeping them busy. They are not able to keep themselves occupied adequately, unless, of course, putting them in front of the TV the whole day.<br>• Well, parents generally are concerned about their children, but, so to say, the fear that the children are too isolated and not seeing their friends again, is greater. But, as I said, they stayed home and whjen schools opened up again, children partly went back to school. However, I have to say, in those cases, when there was a case of COVID in class or parallel class, you said, that is too unsure, I better, no matter what, keep my child at home.<br>• You were not longer allowed to go into the workshop and should stay in the residential facility and you were not allowed to leave your room.<br>• We have handicapped people or impaired children and then the parents had to teach them at home. That is not how it works. One child who needs special education should stay at home and get taught by the mother who has three further children to care for? That is a catastrophe.

Theme	Code	Subcodes	Example quotation[1]
			Even more, if they do not have the right equipment, software and laptop, how is that supposed that work?<br>• I mean, the medical care is working again, that is what we notice, surgeries were made up again which have been postponed, that is working, but the daily care is still difficult.<br>• That has changed, and even worse, with inflation […]. The number of people struggling financially is increasing. We have many members in early retirement or not working at all.
Health Care	Disruption of care	• Medical care<br>• Supportive care<br>• Additional therapies	• In particular, in the first year of the pandemic, patients who were treated in the larger clinics did not get any appointments, because the clinics kept their wards running but did not make any outpatient appointments. So it came up, that some did not see their specialist for more than a year and, if, only on the phone which caused uncertainty.<br>• At first, it was mainly about many people saying 'I isolate myself'. Particularly those with little children who have a nursing service or another kind of support and they have thrown them out at first.<br>• The physiotherapy stopped, the early support stopped, they did not have a chance, it had to be done like that. They tried to do few things online. We thought that is nonsense. […] That was difficult. We saw many people making an effort, but not everything was feasible.
	Nursing services	• Lack of outpatient nursing care<br>• Care assessment by telephone	• Actually one of the biggest problems is, that we do not have any assistants, that there is no longer anyone at the nursing service or that even those families having a personal budget, they simply do not find anyone to hire because there is nobody.<br>• One thing, there surely was, […] there are people in need of nursing care, they always have a care assessment depending on the grade of care you have to be assessed every 6 months or so, also, that the nursing service is working properly not doing any harm. This all was done by phone, which was a relief for some. […]. When the classification actually was done by the medical service, that was by telephone for some time.
	Organization of care	• Higher waiting times<br>• Shorter appointments<br>• Telemedicine<br>• Bureaucracy	• There is one expert center for us in Germany. It is the only clinic for elaborated diagnostics. This was closed temporarily, because the staff was needed to care for the COVID patients. The waiting times were then extended to almost one year […].

Theme	Code	Subcodes	Example quotation[1]
			• Thinking about health care, doctors had little time. Also, which I think, because they had to protect themselves to some extent.<br>• Sometimes they [appointments] were changed into telephone appointments, if it was desired, so you could at least talk to the doctor.<br>•
	Other aspects of care	• Lack of overall healthcare<br>• Possibility of accompaniment in hospitals<br>• Lack of information on rare diseases<br>• Protective measures (e.g. masks, vaccination) in healthcare workers<br>• Triage	• […], what if it happens to me, where can I go to? General practitioners are closing up, so to whom can I go to.<br>• Restrictions of course, it is only allowed to take one accompanying person or no one at all, this is difficult for people who sometimes have intellectual difficulties. This makes the situation more difficult.<br>• It is really difficult for us, because there is no special clinic for our disease, it is too extensive. Therefore, we all – there are a few doctors who have specialized a little – to whom we place. Apart of that there are some families connected to their local practitioners or social pediatric center. That's how it works what is medically. Additionally, we mediate with deaf- and blind-centers. That is spreading a bit, we cannot just look medically, but we have to look in different directions right from the start. […].<br>• Then there was the willingness of professional caregiver, I would say, to wear a mask, was not so great, and then I had the feeling, that someone has been infected with Corona, because the caregivers had taken it a bit easy-going.<br>• Well, what a big topic in our community was, at least in the steering committee was the question of triage […].
Resources	Social Support	• Patient organization<br>• Family and friends<br>• Peers<br>• Functioning (informal) network	• It was more or less the organization, where affected groups could exchange with each other. I think that was a big support.<br>• Those, still having a family, were well off.<br>• They experience, the most severely affected often experience exclusion, the little friends they have are even more important. We notice that, we offer seminars, where people tell, the best thing was to meet again. The topic didn't matter, just to meet and be together in a protected environment, without being looked at stupidly.<br>• […] there is always this team around you, in and around the families, if there were assistants or nurses, the network, what you have built up.

Theme	Code	Subcodes	Example quotation[1]
	Digitalisation	• Online network<br>• Telemedicine	• People who are eighty years and older suddenly got themselves tablets and worked with those and were happy with that. I had an exchange once. The weather was bad and the member said, it is great to meet here and now, we don't need masks and we do not have to do out in this bad weather.<br>• Appointments with the general practitioner were made via telephone, which worked quite well.
	Personality traits/individual characteristics	• Flexibility<br>• Calmness<br>• Optimism and positivity<br>• Ability to self-care (e.g. reduction of information about covid-19)	• We are used that we need to organize ourselves. And then, we prepared ourselves. So you made a little stockpiling, that means, disinfectant, gloves, masks, so you got very creative there.<br>• Maybe, sometimes those highly concerned, a little calmness. If the doctor tells on the telephone, 'ok, there hasn't been anything with your shunt for the last years and now we can skip an appointment and you call me, if somebody is unwell' or something like that. Maybe the one or other family learned to loosen up a bit.<br>• Many [members] have reported that therapies have been cancelled or at least paused, many have also enjoyed that. We had time for ourselves and didn't need to go anywhere every day.<br>• Many [members] have reported that they didn't watch news anymore or no TV at all, only for movies, but no current affairs.
